# Supplementing *Lactobacillus brevis* metabolite modifies carcass performances, characteristics of meat, taste-related compounds and fatty acid profiles of heat stressed Chikso cattle

**DOI:** 10.5713/ab.250444

**Published:** 2025-11-25

**Authors:** Farouq Heidar Barido, Puruhita, Tirtadanu, Kok-Gan Chan

**Affiliations:** 1 Department of Animal Science, Faculty of Animal Science, Universitas Sebelas Maret, Surakarta, Indonesia; 2 Department of Applied Animal Science, Kangwon National University, Chuncheon, Korea; 3 Food Technology of Animal Origin Research Group, Department of Animal Science, Faculty of Animal Science, Universitas Sebelas Maret, Surakarta, Indonesia; 4 Restu Dwi Pangan Co., Tangerang, Indonesia; 5 Research Center for Fishery, National Research and Innovation Agency (BRIN), Bogor, Indonesia; 6 Institute of Biological Sciences, Faculty of Science, University of Malaya, Kuala Lumpur, Malaysia

**Keywords:** Animal Performance, Beef, *GABA*-supplementation, Heat Stress Mitigation, Nutrition, Sustainable Meat Production

## Abstract

**Objective:**

This study aimed to evaluate the effects of purified *Lactobacillus brevis* metabolite (LBMs) supplementation on growth performance, carcass traits, and meat quality in Chikso cattle subjected to heat stress.

**Methods:**

Twenty-seven Chikso steers with an average initial body weight of 499±15 kg were randomly assigned to three treatment groups: a basal diet control (CG), and basal diets supplemented with either 150 mg/kg (LGS) or 300 mg/kg (HGS) of purified LBMs. The feeding trial lasted for 120 days under summer heat stress conditions.

**Results:**

Both LBMs-supplemented groups exhibited significant improvements in average daily gain, with 0.89 g/d (LGS) and 0.81 g/d (HGS) compared to 0.52 g/d in controls (p<0.05). Total weight gain increased by 71% and 56% in LGS and HGS groups, respectively. Meat protein content was significantly higher in LGS (18.97%) and HGS (18.37%) than in CG (17.23%) (p<0.05). Supplementation enhanced water holding capacity and reduced cooking loss to 26.13% from 31.09% in controls (p<0.05). Antioxidant enzyme activities of catalase and superoxide dismutase were elevated in the treatment groups throughout cold storage (p<0.05), with lipid oxidation significantly inhibited from day 3 onward (p<0.05). LBMs also increased oxymyoglobin percentages, indicative of improved meat freshness, and favorably altered fatty acid profiles by decreasing saturated fatty acids and increasing eicosapentaenoic acid levels. Carcass characteristics, however, remained unaffected.

**Conclusion:**

Rumen-protected LBMs supplementation effectively enhances growth performance and meat quality in heat-stressed Chikso steers. These benefits likely arise from improved antioxidant capacity, reduced oxidative stress, and modulation of fatty acid composition. This strategy holds promises for improving livestock resilience to climate challenges and warrants further research.

## INTRODUCTION

Chikso are among four essential and native cattle from Korea, which gained notable exploration as subsequent high-quality meat producer after Hanwoo. The domestic animal diversity information system (DAD-IS) illustrates Chikso as a unique breed, having a brindle-coat like color, while receiving continuously growing demand in the market as safe commodities from indigenous animals [1]. Up to date, studies are being carried out to understand various identified factors, encompassing the environment, genetics, and nutrition on growth performance and carcass characteristics of Chikso breed. Although categorized as a secondary breed for commercial purposes, research indicates that Chikso’s meat yield is comparable to that of Hanwoo, indicated by no notable differences in meat quality, with relatively more abundant volatile compounds for flavour development, like nonanal, octanal, heptanal, and butanal of the aldehydes [2,3].

Among unavoidable factors, alleviation of ambient temperature plays a significant role in deteriorating growth performance, meat quality, and overall characteristics of livestock, including Chikso [4]. With continuously stable increment of soil surface temperature at a staggering 0.6°C each year [5], alternative method to diminish heat shock occurrence and its effects within the farm necessitates serious efforts. As reported by studies, heat shock detrimentally affects overall quality of meat commodities pre- and even post-rigor [4,6,7]. The interferences of hormonal and muscle metabolism by heat stress consequently led to the formation of that so called pale-soft-exudative (PSE); dark-firm-dry (DFD) meat, increased production cost due to additional maintenance and raising management, and significant weight loss [8,9]. Main mechanism is believed through the disturbance of thyroid hormone which function to regulate equilibrium metabolic state, removing excessive heat, and maintenance of water and salt homeostasis. Therefore, un-handled heat stress to livestock animals adversely affects the metabolic homeostasis, high feed conversion ratio, and average daily and final weight gain [9,10].

Several approaches are used to address compromised health and reduced productivity in heat-stressed animals, primarily through non-invasive methods, environmental modification, and nutritional management, with empirical studies revealing that feed and supplement intake modifications are the most effective [11–13]. Optimizing feed composition and targeted supplementation have emerged as effective strategies to counteract these adverse effects. Dietary interventions, including antioxidant-rich supplements and functional amino acids, support the animal’s endogenous defense mechanisms by enhancing oxidative stress mitigation and immune resilience. In addition, these antioxidant-composing amino acids, including arginine, leucine, cysteine, methionine also help reduce the negative effects of secondary metabolite production caused by lipid oxidation and protein denaturation. Furthermore, our earlier work found that elevated antioxidant enzyme activity in essential amino acid-treated hanwoo steers improved surface color, water holding capacity, and organoleptic approval [13].

Lactic acid bacteria (LAB) are increasingly studied for their capacity to produce physiologically beneficial metabolites. Strains specific bacteria including *Lactobacillus brevis* are mainly used to generate metabolites that enhance the immune system in animals. This LAB strain exerts functional effects, leading to both behavioral and physiological improvements in a wide range of animals. One such *L. brevis* metabolite (LBM) is γ-Aminobutyric acid (GABA), a bioactive compound derived from these bacteria. GABA functions primarily as a neurotransmitter inhibitor, promoting health equilibrium in calves and exhibiting protective effects against neurotoxicant-induced cell death. In addition, these four carbon non-amino acid compounds, made from reaction involving glutamic acid serve essential function in maintaining metabolic rates and regulating body temperature of mammals [13,14]. Recent empirical study inferred that LBMs, including exopolysaccharides, short-chain fatty acids (FAs), GABA, and bacteriocins have immunomodulatory activity and can lead to improvements in the immune system [14,15].

Various approaches have been carried out in ascertaining beneficial influence from LBMs, particularly GABA inclusion within diet to enhance growth performance and animal production under conditions of heat stress. Administration to chicks under excessive exposure of heat resulted in the reduction of painting, low dry matter intake, and detrimental quality deterioration caused by heat shock [14,16]. Further, its utilization led to a notable enhancement in production performance and retention of dry matter intake and feed conversion ratio of heat stressed laying hen [17]. While in ruminants, their effect was seen through the retention of carcass performance and meat quality during high-ambient temperature raising conditions [13,18]. It is extensively shown that providing ruminant animals with LBMs, especially GABA is beneficial for supporting animal welfare along with maintenance of efficient livestock raising management. Nevertheless, there has been limited research investigating the impact of supplementing GABA-produced from *L. brevis* on Chikso steers, currently developing and promising beef-producing cattle. With the above premises, this research aimed to extensively understand the influence from supplementing LBM, particularly GABA at low and high parts per million (ppm) on growth performance, carcass profiles, and antioxidative capacity of Chikso Beef reared under heat stress.

The dietary inclusion of metabolites from *L. brevis*, specifically GABA, has garnered considerable attention for its potential to alleviate the detrimental effects of heat stress on animal performance. Empirical evidence in poultry demonstrates that GABA supplementation mitigates heat stress-induced anorexia, behavioral distress, and physiological decline, thereby enhancing growth performance and egg production under thermal challenge conditions [14,16,17]. In ruminants, similar supplementation has shown efficacy in maintaining carcass yield and meat quality despite exposure to elevated ambient temperatures [13,18]. A critical factor underlying the efficacy of these bioactive compounds in ruminants is their delivery in rumen-protected form. Rumen protection safeguards these metabolites from extensive microbial degradation within the rumen environment, ensuring their bioavailability for absorption in the lower gastrointestinal tract, thus maximizing systemic antioxidant capacity and metabolic regulation. This targeted delivery is paramount for optimizing antioxidant amino acid function, such as that of GABA, which supports redox homeostasis, immune resilience, and overall animal welfare during heat stress. Research remains scarce on the effects of rumen-protected GABA derived from *L. brevis* in Chikso cattle an indigenous breed exhibiting significant promise for sustainable beef production. This study seeks to elucidate the influence of dietary supplementation with rumen-protected LBMs, focusing on differential GABA concentrations, on the growth metrics, carcass composition, and antioxidative status of Chikso steers subjected to heat stress.

## MATERIALS AND METHODS

### Biosynthesis and purification of *Lactobacillus brevis* metabolites

LBMs biosynthesis in *L. brevis* proceeds predominantly through the glutamate decarboxylase (GAD) pathway, a mechanism intrinsically linked to acid resistance in bacteria. The process entails the enzymatic conversion of L-glutamate to LBMs, catalyzed by the GAD enzyme, which relies on pyridoxal-5’-phosphate (PLP) as an essential coenzyme. This process is based on previous protocol with minor modification [15]. *L. brevis*, derived from kimchi, was propagated under anaerobic conditions in de Man, Rogosa, and Sharpe broth (Becton-Dickinson and Company, Difco, with pH adjustment to 5.0) with a 5% addition of monosodium glutamate. Incubation proceeded at 32°C for 48 h. The resulting *L. brevis* biomass (designated LBMs) reached an optical density of 1.8 at 600 nm. This corresponded to a viable cell count of 1.0×10^7^ colony-forming units (CFU)/mL, subsequently the clarified supernatant undergoes a range of purification using a high-performance liquid chromatography (HPLC), to purely refine GABA and obtained a concentration of 45 mg/mL.

In addition, in order to strengthen reproducibility and mechanistic interpretation, the LBMs used in the feeding trials were quantified for its GABA content, enabling transparent estimation of the intestinally available GABA. The GABA quantification method exhibited excellent linearity with an r^2^ of 0.99 across the calibration range of 0.05–10 μg/mL. Limits of detection and quantification were 0.06 μg/mL and 0.17 μg/mL, respectively. Recovery rates averaged 98.50±2.30 (n = 5), with intra-day and inter-day precision relative standard deviations (RSDs) of 3.30% and 4.10%, respectively. *In vitro* rumen simulation indicated a rumen bypass fraction of 56.23%±4.84 (±RSDs 8.5%, n = 4) and intestinal release of 78.32%±6.54 (±RSD 8.31%, n = 4) of the bypassed fraction, resulting in an estimated intestinal GABA exposure of 35.12±3.2 mg/kg body weight/day at the 150 mg/kg LBMs dose and 70.21±6.43 mg/kg for high supplementation at 300 mg/kg (data not shown).

### Animals, dietary treatment, and sampling

Twenty-seven Chikso steers, averaging 24±0.76 months in age and 498.98±15 kg in weight were raised at the livestock research institute’s feedlot farm in Gangwon. Chikso steers were assembled into three different sets and housed in three separate enclosures, each measuring 5×10 meters, wherein each treatment has three replications. Each enclosure contained nine steers, which were fed customized diets tailored to their body weights over a 120-day period. During experiments, all animals are exposed to hot weather during summer in Kangwon province, reaching approximately ≥37^o^C and humidity of 77%. This condition is included as moderate stress according to temperature and humidity index (THI). Beyond this THI range, cattle show more pronounced physiological strain, reduced feed intake, and decreased productivity. The diet formulation adhered to standard guidelines published in relevant literature. The control treatment group (CG) received a basal diet consisting of 1.75 kg of rice straw and 10 kg of concentrate daily, containing total digestible nutrients (TDNs) at 74% with 12% protein content. Meanwhile in two intervention treatment groups: low LBMs supplementation (LGS) received a CG incorporated with 150 mg/kg of purified LBMs, while high LBMs supplementation received a CG with inclusion 300 mg/kg of purified LBMs. After the 120-day trial, Chikso were euthanized to measure LBMs influence on essential traits including growth performance, meat quality, carcass characteristics (according to Korean Meat Grading System), and antioxidative profiles. *Longissimus lumborum* from each Chikso beef was packaged into sterile polyethylene (PET) zipper bags within 24 hours postmortem. The samples were stored at 2°C±2°C in a cooling chamber overnight. Nine steaks (2 cm thick) were cut from the strip loin of a single animal. In evaluating quality changes during cold storage, three meat samples from each Chikso beef were assigned into styrofoam, covered with thin plastic wrap, they were put in the dark and refrigerator condition at 4°C±0.2°C for three, six, and nine days. The evaluation of meat proximate, cooking loss, FAs profiles, and other meat quality parameters were conducted on day 2 postmortem using cuts from the previous day. Conditioning samples under vacuum at −24°C and thawed overnight before analysis were carried out for FAs parameters.

### Assessment of meat quality

Proximate analyses, which composed of Soxhlet extracted fat, crude ash, moisture percentage, and protein content were determined according to the AOAC International [19]. The results represent the average of three replicated samples. Instrumental meat color assessment occurred immediately after blooming (exposure of newly sliced meat to oxygen), one-hour post-slicing. Luminosity (CIE L*), yellowness (CIE b*), and redness (CIE a*) values were captured for each sample using a CR-400 Konica Minolta Sensing colorimeter. Calibration of the 8 mm opening of the illuminant light source C, viewed by a 2° observer, was performed using a white calibration plate with color values Y = 93.6, X = 0.3134, and y = 0.3194. Each sample underwent five measurements.

The WHC was evaluated cited the method by [20] using 5 grams of beef samples. Briefly finely ground samples were placed into a centrifuge tube, sealed, and heated in a water bath at 75°C for 30 minutes. The tube was subjected to cooling in iced water for 30 minutes, subjected to centrifugation for 10 minutes under 980×g rotation speed and 24°C temperature. Subsequently, the WHC determination along with moisture content were determined according to AOAC method. In this study, cooking loss was calculated by initially weighing the samples (W1) at 70±5 g. After triplication, beef samples were enclosed in PET zipper bags and submerged into 80°C water for 45 minutes. Submerged beef samples were subjected to cooling at 2°C±2°C for another 30 min and reweighed (W2). Cooking loss percentages were obtained after comparing the weights before and after cooking. Evaluated samples were further utilized to assess the tenderness (hardness) of the beef by the execution of the Warner-Bratzler shear force test utilizing the TA-XT2i Plus instrument (Stable Micro Systems). Following established procedures [21], the meat was sliced into samples measuring 1.5 cm×1 cm. These samples were then placed beneath the V-shaped blade and cut at a consistent speed through the gap in the instrument’s table. The assay parameters used were as follows: pretest speed of 2.0 mm/s, test speed of 1.0 mm/s, and posttest speed of 10 mm/s. The experiment was replicated eight times for each sample.

### Antioxidant enzyme

Quantification of endogenous antioxidant enzymes, catalase (CAT), superoxide dismutase (SOD), and glutathione peroxidase (GSH-Px) were deterimed following reproduceable procedures by [22]. The homogenous beef samples were vigorously mixed with phosphate buffer (pH 7.0 at 25°C), mixed with 2.9 mL of 30 mM H_2_O_2_. The CAT was quantified in units per gram of the sample, with absorbance at 240 nm recorded every 10 seconds over a 2-minute period. SOD was determined during storage using the pyrogallol autoxidation method, with slight modifications as previously described. For SOD, the absorbance at 420 nm were captured every 15 seconds for 2 minutes, compared to tat od standard solution containing 3.025 mL of 50 mM Tris-cacodylate-DTPA buffer, 50 μL of filtered supernatant, and 50 μL of 24.8 mM pyrogallol. Meanwhile, the parameter for GSH-Px was expressed as one unit per grams of sample.

### Lipid oxidation and myoglobin concentration

The thiobarbituric acid reactive substances (TBARS) protocol was employed to quantify lipid oxidation occurrence during storage. An antioxidant mixture weighing 0.1 g, composed of 3% butylated hydroxyanisole, 40% Tween 20, 54% propylene glycol, 3% butylated hydroxytoluene was carefully added to a TBARS test tube at 25 mL size. One percent of TBA in 0.3% NaOH (3 mL) was subsequently added, followed by homogenization along with the 0.5-gram sample 2.5% of TCA in 36 mM HCl (17 mL). Subsequent step was boiling to 100°C for 30 minutes in a water bath, tap water cooling for 15 minutes, and an aliquot of 5 mL aqueous sample was pipetted to a new 15 mL centrifuge tube. The addition of chloroform (3 mL), followed by centrifugation for 30 minutes at 4°C temperature and 2,400×g rotation speed was performed to eliminate impurities. MDA content was evaluated at 532 nm using a UV spectrophotometer against a blank containing distilled water. The results were expressed as milligrams of malondialdehyde per kilogram of sample. Each analysis was repeated four times.

In parallel, myoglobin alteration state in Chikso beef during storage were monitored by mixing a 1-gram sample with sodium phosphate solution (0.04 mol/L, pH 7) (8 mL). After vigorous shaking, the samples were brought into centrifugation for 15 minutes at 10,000×g rotation speed. Filtration using Whatman paper No. 56 was carried out, continued by absorbance measurement at 525, 545, 565, and 575 nm using a UV-spectrophotometer, with a blank containing distilled water for reference. The documented wavelength was used to calculate the percentages of deoxymyoglobin (DMb), oxymyoglobin (OMb), and metmyoglobin (MMb).

### Fatty acid composition

The composition of FAs was determined using an Agilent gas chromatography instrument, coupled with an automated sampler. A chloroform-methanol solution (2:1 v/v) was used to extract the fat from meat, following the method described in by [23]. Prior injection to GC-system, esterification of FAs into methyl esters, followed by dissolving into 1.5 mL of hexane was carried out. A total of 1 μL of each sample was injected into GC-system through the automated sampler, adhering to the specified injection parameters. The split ratio was set at 100:1, and the injector temperature was held at 250°C. A wall-coated open tubular (WCOT) capillary column made of fused silica was used to separate the FA methyl esters. This column had a 0.25 mm internal diameter, a length of 100 m, and a film thickness of 0.20 μm. The flow rate of helium as the carrier gas was 1.0 mL/min. The oven temperature program started at 150°C for 1 minute, increased by 7°C per minute to 200°C, held for 5 minutes, then increased by 5°C per minute to 250°C, and finally held at 250°C for 10 minutes. The detector temperature was set at 275°C. The FAs were identified by comparing the retention times of the sample peaks with those of FAs standards (47015-U; Sigma-Aldrich). The percentage of each FAs was calculated based on the peak area relative to the total peak area of all identified FAs.

### Statistical analysis

The animal performance data, including repeated measures of average daily gain (ADG), were analyzed using linear mixed-effects models with treatment, time, and their interaction as fixed effects. Initial body weight was included as a covariate, where animal identity was treated as a random effect to account for repeated measurements within subjects. The covariance structure was selected based on the lowest Akaike’s information criterion (AIC). Models were fit by restricted maximum likelihood (REML). Post hoc comparisons were performed using Tukey’s HSD test, where statistical significance was determined at p<0.05. The linear mixed model of two-way analysis of variance (ANOVA) was also employed to determine the significance of both HGS and LGS to determine meat quality parameters throughout cold storage experiments. In addition, the time-point data for carcass traits, proximate composition, cooking loss, water holding capacity, FA profiles, and shear force were evaluated by one-way ANOVA using the Agricolae package in R v3.6.1 (The R Foundation for Statistical Computing). Normality and homoscedasticity were previously verified, with data transformations applied when necessary. Pairwise group differences were assessed via Duncan multiple range test, with significance defined at p<0.05.

## RESULTS AND DISCUSSION

### Growth performance and carcass characteristics

The nutritional quality of beef stems from intricate factors including raising management, proper slaughtering techniques, and post-mortem handling, with ante-mortem management being crucial for determining meat quality [2]. As global temperatures rise and demand for healthier, safer meat increases, significant efforts focus on incorporating substances with high biological functions during the raising phase. These substances positively affect the intestinal system, immunoregulatory responses, and enhance endogenous enzyme activity, leading to improved animal performance, carcass conformation, and overall meat quality [4].

In this study, LBMs were incorporated at low and high concentration in Chikso steers’ diets to minimize destructive effect from heat stress. It is validated that heat stress detrimentally affect meat quality through metabolic imbalances, resulting in enzymatic and hormonal activity interference [22]. Further, catecholamines and corticosterone are produced as a cellular response against heat shock. Whereas the secretion of these enzymes leading to the occurrence of lipid peroxidation and destruction of immunoregulatory leucocytes, mainly type B and type T cells. This research incorporated purified LBMs containing various bioactive compounds, mainly the GABA. Both supplementation of LBMs at low and high concentrations into the diets of Chikso steers to mitigate detrimental effects caused by heat stress. He et al [24] has reported that the attachment of peroxyl radicals from lipid oxidation process to proteins disrupts calpain activity, consequently diminishing myofibrillar fragmentation and resulting in a stiffer and tougher meat texture.

Table 1 illustrated notable influence from LBMs supplementation on growth performance of Chikso. Succeeding 120 days of raising under summer temperature, averagely at 37°C and relative humidity of 67.02%, the ADG was differed significantly among treatments. Recorded ADG was the highest under LGS with 0.89 g/d, followed by HGS with 0.81 g/d, and control at 0.52 g/d (p*<*0.05). Similar pattern was also documented for total gained kg, with 106.27, 97.26, and 62.11 kg for LGS, HGS, and Control, respectively (p*<*0.05). Yet, LGS and HGS shared no differences, indicating absence effect from extended concentrations. Regarding the carcass characteristics, this study found no notable influence from LBMs supplementation (p*>*0.05). A detailed record for carcass profiles is displayed in Table 2.

The study demonstrated a significant incremental improvement in weight gain and ADG of Chikso, independent of LBMs concentration. While LBMs supplementation had positive effects, increasing its concentration in the diets did not yield significant changes. On the other hand, GABA, present in the mammalian neurotransmitter system, typically acts to counteract increased cortisol secretion during stress, wherein excessive stress can decrease GABA activity, leading to diminished behavior and performance in animals [18]. The inclusion of LBMs, with targeted presence of GABA in this study aimed to enhance its availability, even under stress. Tang and Chen [16] indicated that LBMs supplementation in broiler diets helps mitigate heat stress effects and maintain feed intake by increasing the secretion of digestive and endogenous antioxidant enzymes, as well as stabilizing the body’s electrolyte balance. Further, the mechanism of relaxation regulated by the GABAergic receptor helps the animals’ cellular environment to retain at a relaxed state even under stressed condition. Nevertheless, the higher score for expected parameters in carcass characteristics and animal performances were not seen in this study using ruminant, which also confirmed previous study in Hanwoo [13] and Jinjiang Yellow Cattle [10], which also reported no significant effect from increased LBMs concentration on yield traits. This helps to infer a limited effect from dietary LBMs supplementation to carcass profiles of the cattle.

### Meat quality

**Figure 1:**
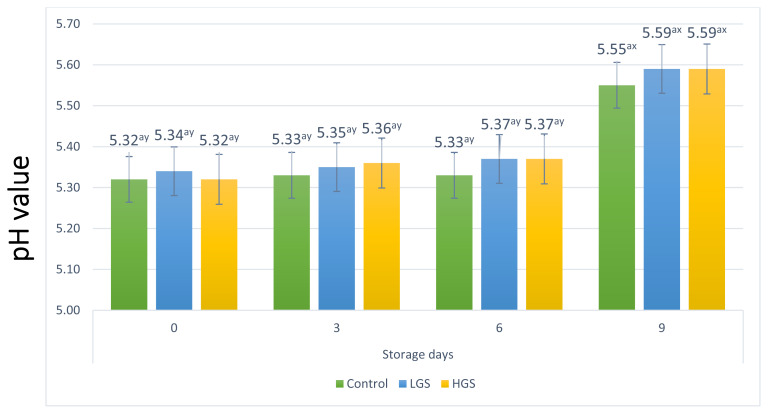
Evaluation of *longissimus lumborum’s* pH from Chikso steers following LBMs supplementation during 9 days cold storage. The data were taken with 3 days interval from prior measurement. Control, basal diet with 74% total digestible nutrient and protein level at 12%; LGS, CG supplemented with LBMs at 150 mg/kg; HGS, CG supplemented with LBMs at 300 mg/kg. All data were represents as means value. No modification from LBMs supplementation on meat pH, while documented significant increment at ultimate storage day (day 9) in comparison to day 0, 3, and 6 (p<0.05). ^a,b^ Means within each graph indicates significant influence from LBMs treatments; ^x,y^ Means across the graph indicates significant different as influenced by storage time LBM, *Lactobacillus brevis* metabolite.

The utilized samples were characterized as grade 1 according to the Korean meat grading system, and the meat quality resulted from LBMs administration are displayed in Table 3. Protein content experienced notable increment, with LGS (18.37%) and HGS (18.97%) shared similarly higher percentages compared to control. Total water content, Soxhlet extracted fat, and crude ash percentage experienced no alteration even after high LBMs supplementation (p*>*0.05). Further, positive influence from supplementing Chikso cattle with LBMs are seen from markedly higher water retained after cooking, with HGS treated groups had the lowest cooking loss (26.13%), in comparison to LGS (30.47%) and control (31.09%) (p*<*0.05). No extended effect was recorded regarding to its WHC and shear force value (p*>*0.05). In addition, regarding the meat pH during storage days (Figure 1), this study obtained no modification from LBMs supplementation, while documented significant increment at ultimate storage day in comparison to day 0, 3, and 6 (p*<*0.05).

The study revealed that LBMs supplementation positively affects the synthesis of protein in Chikso beef, with both high and low concentrations of LBMs showing significantly higher percentages compared to the control. Zhang et al [25] observed an enhanced efficiency and increased biological availability in the rumen of animals administered with GABA. This could be attributed to the heightened efficacy and presence of protein-digestive enzymes like trypsin, which aid in protein synthesis. Additionally, these findings align with recent research that reported consistent feed intake under heat stress and improved nutrient absorption efficiency, owing to greater gastrointestinal effectiveness and enhanced digestibility of crude protein, fat, and calcium [26]. Moreover, cooking loss has been observed to improve with LBMs supplementation, but only at high dietary concentrations. This parameter is known to have a negative correlation with standard meat quality and antioxidant capacity, reflecting the meat muscle’s ability to retain water within the muscle fiber environment. Kinara et al [27] and Barido et al [28] highlighted the beneficial impact of antioxidants and amino acids rich in antioxidants on maintaining the desired quality of meat. These substances protect the integrity of cell membranes against damage from prooxidant activities. The formation of free radicals through the oxidation and denaturation of lipids and proteins can alter membrane conformation by disrupting the synthesis of lipoproteins, lipids, and other components involved in cellular membrane regeneration that cause a membrane permeability to increase [22,29]. Additionally, GABA plays a dominant role in regulating the cellular equilibrium of electrolytes, which impacts the muscle’s capacity to retain water during the processing phase. This observation is corroborated by an increase in WHC at higher concentrations. This aligns with the research conducted by [24,29], which demonstrated reduced cooking shrinkage following the supplementation of cattle diets with antioxidant-rich oils.

### Antioxidant enzyme and lipid oxidation

The antioxidant capacity from endogenous enzymes, particularly CAT, GSH-Px, and SOD during cold storage are presented in Table 4. The dietary treatment of LBMs both at low and high concentration to Chikso promoted the more availability of CAT and SOD unit/g meat throughout storage days (9 d). The CAT was recorded the highest at initial storage day for LGS (335.30 unit/g meat) and HGS (337.21 unit/g meat), and started to markedly weaker after storage day 6, and 9. SOD shared no different trend, with LGS and HGS exhibited activity at 50.26 and 50.11 unit/g at day 0, while showed notably lower value at day 6, and day 9. Further, no notable influence from supplementing LBMs to the availability of GHS-Px enzyme throughout 9 days of cold storage (p*>*0.05). This present study confirmed that LBMs, in both low and high concentrations within cattle diets, enhanced the activity of CAT and SOD enzymes. These enzymes are crucial in neutralizing excessive free radicals through hydrogen donation and scavenging mechanisms, providing cryoprotective protection for cells against the harmful effects of lipid oxidation and protein denaturation [30]. Further, the result demonstrated that LBMs supplementation positively affected CAT and SOD activities, with limited impact on GSH-Px enzymes, suggesting an increase in antioxidant capacity. The improvement mechanism, as described by [17], involves the enhanced conversion efficiency and regulation of glutamic acid into vital endogenous antioxidant enzymes, including GSH-Px, SOD, and CAT. These findings corroborate previous research in cows [18], lamb [31], goat meat [32] and cattle [13], in which aggregation of antioxidant containing substances enhances endogenous antioxidant enzymes within the blood system. In addition, this study indicates LBMs’ effectiveness in scavenging an overproduction of reactive oxidative species in the form of hydroxyl radical, singlet oxygen, and diatomic oxygen under excessive exposure of heat.

**Figure 2:**
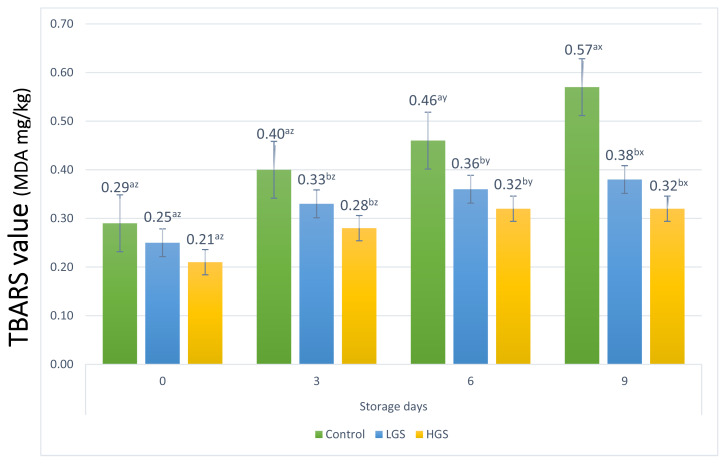
Evaluation of *longissimus lumborum’s* lipid oxidation, measured using TBARS evaluation assay on Chikso steers following LBMs supplementation during 9 days cold storage. The data were taken with 3 days interval from prior measurement. Control, basal diet with 74% total digestible nutrient and protein level at 12%; LGS, CG supplemented with LBMs at 150 mg/kg; HGS, CG supplemented with LBMs at 300 mg/kg. All data were represents as means value. Both LGS and HGS supplementation shown to effectively suppress MDA formation, resulting a lower lipid oxidation occurrence throughout cold storage, in comparison to control (p<0.05). ^a,b^ Means within each graph indicates significant influence from LBMs treatments; ^x,z^ Means across the graph indicates significant different as influenced by storage time. TBARS, thiobarbituric acid reactive substances; LBM, *Lactobacillus brevis* metabolite.

This present study utilized TBARS assay in measuring lipid oxidation rate and expresses it as the amount of MDA in mg/kg meat. LGS and HGS groups were shown to effectively suppress the formation of MDA, resulting from lipid oxidation occurrence throughout cold storage, in comparison to CG (p*<*0.05) starting from day 3 and continuing until the end of storage period (day 9) (Figure 2). Nevertheless, the lipid oxidation rate in the group that received a greater supplementation of 300 mg/kg did not show any significant difference compared to that of low ones 150 mg/kg (p*>*0.05). Further, the ANOVA did not reveal any significant interactivity between treatment and storage period.

The TBARS analysis, which quantifies the concentration of lipid oxidation products, revealed significantly lower MDA levels in LBMs-treated beef from day 3 onwards, persisting throughout the storage period. This finding aligns with the observed increase in antioxidant enzyme activity, affirming the enhanced antioxidative capacity attributed to LBMs supplementation. Furthermore, the study noted a substantial rise in OMb percentages, a direct indicator of meat freshness, and a reduction in meat discoloration. The preservation of OMb is advantageous during cold storage as it is associated with a bright cherry red color indicative of freshness. Conversely, a higher proportion of MMb suggests increased lipid oxidation, an occurrence not detected in LBMs-treated samples. Xu et al [33] stated that the oxidative status of meat reflects the health of the animal prior to the sacrifice. Meanwhile, the lipid oxidation adversely affects meat quality in various ways, including during rigor mortis, where it slows myofibrillar degradation, leading to reduced WHC and a tougher meat texture. Moreover, the excessive production of MDA from lipid oxidation can hasten meat spoilage by accelerating color discoloration. Subsequently, the addition of antioxidants can mitigate these adverse effects. Therefore, this study inferred that LBMs supplementation in this study signifies an improvement in the quality of Chikso beef during cold storage.

### Myoglobin and instrumental color

The measurement system of CIE was utilized to measure luminosity (L*), redness (a*), and yellowness (b*) from Chikso meat after LBMs supplementation (Table 5). There was no distinction in discoloration rate until day 3, wherein CIE a* under LGS and HGS retained markedly higher value compared to control (p*<*0.05). In contrast, CIE b* was notably higher at day 6 and 9 under control treated groups (p*<*0.05). Again, no distinction was obtained from supplementing different ppm of LBMs to Cikso. Cold storage notably decreased the CIE b* of Chikso beef regardless of treatment groups.

Alteration state of myoglobin percentages, composed of OMb, DMb, and MMb over storage period was illustrated in Table 6. The storage duration had a substantial impact on the profiles of myoglobin, particularly OMb, DMb, and MMb profiles (p*<*0.05). A statistical significance was noted on OMb between CG and LBMs treatments. LBMs-treated samples exhibited a greater percentage of OMb, starting from day 3 of storage, up to the ultimate storage day. Nevertheless, the extent of LBMs doses within cattle’s diet did not do much in determining OMb, implying efficient application of LGS. Equivalent findings were also achieved for the proportion of MMb. The MMb production is correlated with meat discoloration, with a greater proportion indicating a higher incidence of discoloration. In addition, Table 6 demonstrates an irregular trend in the development of MMb following supplementation. On the third day, CG had a larger proportion of MMb production compared to LBMs-treted groups. The percentages were 38.47%, 32.84%, and 32.17% for CG, LGS, and HGS, respectively. No discernible variation was recorded on day 6 of cold storage. This study suggested the supplementation of LBMs to provide substantial influence on the diminish production of MMb. LBMs supplementation inhibited the MMb synthesis and favorably contributed to maintaining a higher proportion of OMb during cold storage. However, the increased dosage of LBMs did not result in a significant improvement of meat freshness as indicated by no difference in OMb and MMb during cold storage.

### Taste-related compounds and equivalent umami concentrations

In this study, the equivalent umami concentration (EUC) was quantified to assess the extent of influence from LBMs supplementation on umami formation in Chikso beef. Numerous studies have employed this variable to demonstrate changes in taste-related compounds, as it is an effective method to measure umami concentration, resulting from the synergistic interaction among various umami-associated compounds, specifically the 5’-nucleotides, and glutamic and aspartic acids [11,17,34]. The quantification results, obtained through HPLC analysis of beef samples post-LBMs supplementation, are presented in Table 6. High levels of LBMs supplementation resulted in meat with a greater intensity of glutamic acid, as indicated by a significantly higher value (0.69 g/kg) compared to the control (0.57 g/kg). However, no significant differences were observed between low and high LBMs supplementation, with values of 0.61 and 0.69 g/kg for LGS and HGS, respectively. Additionally, no notable differences were detected for other variables such as 5′-AMP, 5′-IMP, 5′-GMP, and aspartic acid, suggesting a limited effect of LBMs supplementation on the formation of these compounds (p*>*0.05).

Regarding to the influence of LBMs to taste-related compounds of Chikso beef, although clear mechanisms are rarely accessible, it is assumed that the intensification in glutamic acid levels following LBMs supplementation in this present study is the result of multiple biochemical pathways. LBMs is produced from glutamate by the enzyme GAD, and introducing LBMs could alter neurotransmitter metabolism, possibly raising glutamate’s availability for synthesis. Meanwhile, GABA as the main purified product from LBMs as an inhibitory neurotransmitter, possibly act to modulate neuronal excitability and prompt a greater release of glutamate in muscle tissues via feedback loops. In addition, the provision of GABA within the animal’s diet may also impact and stimulate more diverse composition and population of gut microbiota, influencing the amino acid metabolism. A positively impactful or a healthier gut microbiome can improve the absorption and metabolism of dietary amino acids, including glutamate. Another possible leveraging mechanism could also from the enhanced nutrient utilization from LBMs that lead to the increased levels of essential amino acids in meat products. Additionally, the interaction of umami-related compounds like 5’-nucleotides with LBMs could further boost glutamic acid concentrations, intensifying the umami taste in beef [3,17].

### Fatty acid profiles

The alteration of FAs resulting from LBMs supplementation to Chikso are displayed in Table 7. Significant reduction was observed on saturated fatty acids (SFAs) (p*<*0.05), mainly the myristic (C14:0), palmitic (C16:0), and stearic (C18:0) acids (p*<*0.05). HGS supplementation shared similar effect on C14:0 and C16:0 with LGS, while yielding the lowest reduction against C18:0 (p*<*0.05). Besides, LBMs supplementation did notable modification on individual poly unsaturated fatty acids (PUFAs), particularly eicosapentaenoic acid (EPA) (C20:5n3), in which both LGS and HGS had notably higher concentrations in comparison to control. However, total PUFAs were not changed regardless of treatment groups. Further, no differences were inferred on total SFAs, monounsaturated fatty acids (MUFAs), and PUFAs in Chikso beef after low or high LBMs supplementation (p*>*0.05).

The composition of FAs is crucial to the quality of beef, significantly influencing antioxidant profiles, flavor, and consumer acceptability. This study highlights the role of LBMs in modifying FAs of Chikso beef. The supplementation of LBMs to Chikso’ diet was empirically reduced the proportion of individual SFAs, especially C14:0, C16:0, and C18:0, supporting findings by [24] and [35], who noted a decrease in stearic and palmitic acids with antioxidant-rich supplements like oregano and linseed oils. Proposed mechanism is presumably through the ability of bioactive compounds from plant sources and metabolites from purified microorganisms to enhance nutrient absorption and modify the cellular pathways for intramuscular fat distribution, as noted by [36]. In addition, [37] described how dietary antioxidants specifically regulate the biohydrogenation of C18:0, altering the proportion of SFAs in beef. Besides, this study also reports an increase in EPA, likely due to the heightened activity of delta 5 and 6 desaturases, enzymes that convert alpha-linolenic acid into EPA. This aligns with findings by [24,35] who reported enhanced PUFA content following dietary supplementation with essential oils and antioxidants. Additionally, the desaturation and elongation processes contribute to reducing the risk of diabetes and cardiovascular diseases by lowering blood pressure and lipid levels. Ultimately, this study showed a positive influence from LBMs supplementation to reduction of omega 6 to omega 3 ratio, wherein both LGS and HGS maintained a ratio of 8.87:1 and 8.94:1, respectively, while CG recorded at 10.65:1. Reduction of omega 6 to omega 3 ratio in meat is preferable due to its beneficial effect to health by inhibiting atherosclerosis, occurrence of blood pressure and cardiovascular diseases. However, ratio of not exceeding 11:1 is confirming normal level in grain fed cattle. The individual FAs identified in this study are consistent with previous research [9,13,24], which found that the predominant FAs include oleic and palmitoleic acids (MUFAs), linoleic, alpha-linolenic, arachidonic acids, and EPA (PUFAs), along with palmitic and stearic acids (SFAs).

## CONCLUSION

The supplementation of purified LBMs, with the main composition of GABA in Chikso cattle diets has demonstrated significant benefits for animal performance and overall meat quality, particularly in heat stressed Chikso. Our findings indicate that both low and high doses of LBMs markedly improved ADG and overall weight gain, while inhibiting the excessive occurrence of lipid oxidation. The LBMs supplementation upregulates endogenous antioxidant enzymes, particularly CAT and SOD, creating a steadily stable color and minimize meat discoloration. This study showcasing the potential of LBMs as an effective nutritional strategy for enhancing livestock productivity.

## Figures and Tables

**Table 1:** Animal performance of Chikso steers as influenced by the inclusion of LBMs

Parameters	Treatments			SEM	p-value

	CG	LGS	HGS		
Animal performance					
Initial weight (kg)	457.02	460.01	451.97	11.08	0.71
Final weight (kg)	519.13	566.28	549.23	15.22	0.12
Weight gain (kg)	62.11^b^	106.27^a^	97.26^ab^	3.52	<0.05
ADG (g/d)	0.52^b^	0.89^a^	0.81^a^	0.07	<0.05
Feed formulation					
Concentrate (kg)	10	10	10	-	-
LBMs (mg/kg)	-	150	300	-	-
Straw (kg)	1.75	1.75	1.75	-	-
Total (kg)	11.75	11.75	11.75	-	-

CG, basal diet with 74% total digestible nutrient and protein level at 12%; LGS, CG supplemented with LBMs at 150 mg/kg; HGS, CG supplemented with LBMs at 300 mg/kg.

a,b Means within each row indicates significant influence from LBMs treatments.

LBM, *Lactobacillus brevis* metabolite; SEM, standard error of the mean; ADG, average daily gain.

**Table 2:** Carcass profiles from Chickso steers as influenced by the inclusion of LBMs

Parameters	Treatments			SEM	p-value

	CG	LGS	HGS		
Yield traits^1)^					
Hot carcass weight (kg)	311.48	339.77	329.54	21.95	0.51
Backfat thickness (mm)	65.21	70.14	70.09	4.19	0.09
Rib eye area (cm^2^)	10.04	11.41	10.97	2.06	0.17
Yield index^2)^	4	4	4	0.29	0.51
Yield grade^3)^	59.88	59.13	60.07	1.09	0.39
Quality traits	3	3	3	0	0.38
Marbling score^4)^	3	3	3	0	0.6
Meat color^5)^	1.3	1.6	1.6	0.2	0.29
Fat color^6)^	2.5	3.2	3	0.19	0.35
Firmness^7)^	1.6	1.1	1.1	0.06	0.12
Maturity^8)^	3	2.7	3.1	0.15	0.27
Quality grade^9)^	329	351	360	20.09	0.25

CG, basal diet with 74% total digestible nutrient and protein level at 12%; LGS, CG supplemented with LBMs at 150 mg/kg; HGS, CG supplemented with LBMs at 300 mg/kg.

1) Area was measured from longissmus thoracis muscle.

2) Yield index was calculated by equation 65.834−(0.393×Backfat thickness [mm])+(0.088×Ribeye area [cm^2^])−(0.008×carcass weight [kg])+2.01.

3) A grade (yield index≥69.00) = 3, B grade (66.00≤yield index<69.00) = 2, C grade (yield index<66.00) = 1.

4) Marbling score standard; No.1–No.9 (1 = devoid, 9 = abundant).

5) Meat color standard; No.1–No.7 (1 = brightly cherry red, 7 = extremely dark red).

6) Fat color standard; No.1–No.7 (1 = white, 7 = dark yellow).

7) Firmness score; 1 to 3 (1 = soft, 3 = firm).

8) Maturity score; 1 to 3 (1 = youthful, 3 = mature).

9) 1++ grade = 5, 1+ grade = 4, 1 grade = 3, 2 grade = 2, 3 grade = 1.

a,b Means within each row indicates significant influence from LBMs treatments.

LBM, *Lactobacillus brevis* metabolite; SEM, standard error of the mean.

**Table 3:** Proximate composition and meat quality from *longissimus lumborum* of Chickso steers as influenced by the inclusion of LBMs

Parameters	Treatments			SEM	p-value

	CG	LGS	HGS		
Mositure	68.46	67.75	66.91	1.04	0.71
Crude fat	13.65	12.88	13.11	1.86	0.53
Crude protein	16.88^b^	18.37^a^	18.97^a^	0.18	<0.05
Crude ash	1.01	1	1.01	0.01	0.21
Water holding capacity (%)	72.09^b^	74.97^ab^	76.36^a^	1.20	0.72
Cooking loss (%)	31.09^a^	30.47^a^	26.13^b^	0.41	<0.05
Shear force (kg)	5.82	5.58	5.42	0.51	0.07

CG, basal diet with 74% total digestible nutrient and protein level at 12%; LGS, CG supplemented with LBMs at 150 mg/kg; HGS, CG supplemented with LBMs at 300 mg/kg.

a,b Means within each row indicates significant influence from LBMs treatments.

LBM, *Lactobacillus brevis* metabolite; SEM, standard error of the mean.

**Table 4:** Antioxidant enzymes of *longissimus lumborum* of Chickso steers as influenced by the inclusion of LBMs

Parameters	Storage (d)	Treatments			SEM	p-value		
	
		CG	LGS	HGS		Diet	Storage	Diet×Storage
CAT (unit/g meat)	0	314.35^bx^	335.30^ax^	337.21^ax^	2.55	<0.05	<0.05	<0.05
	3	307.12^bx^	330.67^ax^	333.07^ax^	3.18			
	6	300.01^by^	320.52^ay^	321.28^ay^	3.03			
	9	289.11^by^	300.11^ay^	297.57^ay^	4.11			
SOD (unit/g meat)	0	46.10^bx^	50.26^ax^	50.11^ax^	0.15	<0.05	<0.05	<0.05
	3	43.12^bx^	49.75^ax^	50.01^ax^	0.09			
	6	39.34^bx^	49.21^ax^	49.06^ax^	0.10			
	9	30.41^by^	40.77^ay^	41.02^ay^	0.27			
GSH-Px (unit/g meat)	0	1.58^x^	1.59^x^	1.55^x^	0.07	0.07	<0.05	<0.05
	3	1.51^x^	1.52^x^	1.56^x^	0.01			
	6	1.42^y^	1.44^y^	1.48^y^	0.05			
	9	1.05^z^	1.10^z^	1.11^z^	0.07			

CG, basal diet with 74% total digestible nutrient and protein level at 12%; LGS, CG supplemented with LBMs at 150 mg/kg; HGS, CG supplemented with LBMs at 300 mg/kg.

a,b Means within each row indicates significant influence from LBMs treatments.

x,y,z Means within the same column indicates significant different as influenced by storage time.

LBM, *Lactobacillus brevis* metabolite; SEM, standard error of the mean; CAT, catalase; SOD, superoxide dismutase; GSH-Px, glutathione peroxidase.

**Table 5:** CIE color and relative myoglobin concentration (%) of *longissimus lumborum* from Chickso steers as influenced by the inclusion of LBMs

Parameters	Storage (d)	Treatments			SEM	p-value		
	
		CG	LGS	HGS		Diet	Storage	Diet×Storage
CIE L*	0	34.63	33.86	34.72	0.92	0.09	0.75	0.55
	3	32.10	32.94	33.72	0.67			
	6	33.84	33.56	34.97	0.84			
	9	34.02	35.88	35.74	0.25			
CIE a*	0	22.37	21.42	22.44	0.45	<0.05	<0.05	0.27
	3	20.56	20.99	21.10	0.89			
	6	22.87	22.55	23.18	0.34			
	9	13.62^b^	16.00^a^	16.73^a^	0.26			
CIE b*	0	12.34	12.38	12.85	0.34	0.65	<0.001	0.43
	3	11.17	11.51	11.89	0.28			
	6	10.87	11.47	11.74	0.15			
	9	9.08	9.42	8.88	0.75			
Oxymyoglobin (%)	0	59.38	60.47	61.37	1.14	<0.05	<0.001	0.47
	3	45.21^b^	49.91^a^	50.22^a^	1.29			
	6	31.71^b^	39.51^a^	39.75^a^	1.25			
	9	15.39^b^	24.27^a^	26.28^a^	2.65			
Deoxymyoglobin (%)	0	26.23	27.59	27.45	1.36	0.959	<0.05	0.44
	3	26.36	26.03	27.27	0.38			
	6	19.35	17.58	18.21	0.99			
	9	18.25	18.69	18.66	0.43			
Metmyoglobin (%)	0	22.95	21.96	21.5	0.73	<0.05	<0.001	0.75
	3	38.47^a^	32.84^b^	32.17^b^	0.19			
	6	34.71	32.98	33.1	0.21			
	9	52.61^a^	45.73^b^	47.85^b^	1.09			

CG, basal diet with 74% total digestible nutrient and protein level at 12%; LGS, CG supplemented with LBMs at 150 mg/kg; HGS, CG supplemented with LBMs at 300 mg/kg.

a,b Means within each row indicates significant influence from LBMs treatments.

LBM, *Lactobacillus brevis* metabolite; SEM, standard error of the mean.

**Table 6:** Nucleotide compounds and equivalent umami concentration (EUC) from *longissimus lumborum* of Chickso steers as influenced by the inclusion of LBMs

Parameters	Treatments			SEM	p-value

	CG	LGS	HGS		
5′-AMP (g/kg)	0.16	0.16	0.17	0.02	0.14
5′-IMP (g/kg)	2.81	2.78	2.80	0.03	0.09
5′-GMP (g/kg)	0.84	0.84	0.83	0.01	0.21
Glutamic acid (g/kg)	0.57^b^	0.61^ab^	0.69^a^	0.05	<0.05
Aspartic acid (g/kg)	ND	ND	ND	0.00	

CG, basal diet with 74% total digestible nutrient and protein level at 12%; LGS, CG supplemented with LBMs at 150 mg/kg; HGS, CG supplemented with LBMs at 300 mg/kg.

a,b Means within each row indicates significant influence from LBMs treatments.

LBM, *Lactobacillus brevis* metabolite; SEM, standard error of the mean; ND, not detected.

**Table 7:** Fatty acid profiles of *longissimus lumborum* from Chickso steers as influenced by the inclusion of LBMs

Parameters	Treatments			SEM	p-value

	CG	LGS	HGS		
C14:0	3.59^a^	3.19^b^	3.15^b^	0.09	<0.05
C16:0	30.09	28.02	26.66	0.49	0.42
C16:1n7	5.50	5.68	5.66	0.04	1.01
C18:0	10.11	9.54	9.27	1.02	0.52
C18:1n9	46.44	46.22	46.37	0.47	1.21
C18:2n6	2.71	2.69	2.71	0.25	0.98
C18:3n3	0.24	0.25	0.26	0.01	0.87
C20:4n6	0.06	0.06	0.06	0.02	1.02
C20:5n3	0.02^b^	0.06^a^	0.05^a^	0.03	<0.05
SFA	43.79^a^	40.75^b^	39.08^b^	1.21	<0.05
MUFA	51.94	51.90	52.03	0.11	0.29
PUFA	3.03	3.06	3.08	0.14	0.16
n-3	0.26	0.31	0.31	0.06	0.32
n-6	2.77	2.75	2.77	0.13	0.19
n-6/n-3	10.65^a^	8.87^b^	8.94^b^	0.97	<0.05

CG, basal diet with 74% total digestible nutrient and protein level at 12%; LGS, CG supplemented with LBMs at 150 mg/kg; HGS, CG supplemented with LBMs at 300 mg/kg.

a,b Means within each row indicates significant influence from LBMs treatments.

LBM, *Lactobacillus brevis* metabolite; SEM, standard error of the mean; SFA, saturated fatty acid; MUFA, monounsaturated fatty acid; PUFA, poly unsaturated fatty acid.

## Data Availability

Upon reasonable request, the datasets of this study can be available from the corresponding author.
